# A New Editor

**Published:** 1883-01

**Authors:** 


### A New Editor.

It is just announced that W. C. Barrett, M.D., D.D.S., M.D.S., of Buffalo, N. Y., on the first of January, 1883, took the entire editorial charge of the dental department of "*The Independent Practitioner*," heretofore edited by Dr. M. B. Wilkerson.

We extend to Dr. Barrett the editorial hand, and best wishes for success in his new field of labor. It is quite certain that Dr. B's utterances will give no uncertain sound. Long may he wave.

